# Investigating the impact of terminal heat stress on contrasting wheat cultivars: a comprehensive analysis of phenological, physiological, and biochemical traits

**DOI:** 10.3389/fpls.2023.1189005

**Published:** 2023-08-30

**Authors:** Hitesh Kumar, Vishal Chugh, Manoj Kumar, Vikas Gupta, Shambhoo Prasad, Satish Kumar, Chandra Mohan Singh, Rahul Kumar, Bhupendra Kumar Singh, Gurusharan Panwar, Mukul Kumar

**Affiliations:** ^1^ Department of Genetics and Plant Breeding, College of Agriculture, Banda University of Agriculture and Technology, Banda, Uttar Pradesh, India; ^2^ Department of Basic and Social Sciences, College of Horticulture, Banda University of Agriculture and Technology, Banda, Uttar Pradesh, India; ^3^ Division of Crop Improvement, ICAR-Indian Institute of Wheat and Barley Research, Karnal, Haryana, India; ^4^ Department of Plant Molecular Biology and Genetic Engineering, Acharya Narendra Deva University of Agriculture and Technology Kumarganj, Ayodhya, Uttar Pradesh, India; ^5^ Department of Plant Science and Landscape Architecture, University of Connecticut, Storrs, CT, United States; ^6^ Department of Entomology, College of Agriculture, Banda University of Agriculture and Technology, Banda, Uttar Pradesh, India; ^7^ Department of Agronomy, College of Agriculture, Banda University of Agriculture and Technology, Banda, Uttar Pradesh, India

**Keywords:** *Triticum aestivum* L, heat stress, canopy temperature, NDVI, chlorophyll, antioxidants, biochemical mechanism

## Abstract

Terminal heat stress has become one of the major threats due to global climate change which is significantly affecting the production and productivity of wheat crop. Therefore, it is necessary to identify key traits and genotypes to breed heat-tolerant wheat. The present study was undertaken with the objective of comparing the effects of heat stress (HSE) and extended heat stress (EHSE) on phenological-physio-biochemical traits of contrasting heat-tolerant and heat-susceptible genotypes during the reproductive phase. Phenological traits exhibited significant reduction under EHSE compared to HSE. Heat-tolerant genotypes maintained balanced phenological-physio-biochemical traits, while heat-sensitive genotypes showed significant reductions under both stress regimes. Among phenological traits, DM (R^2^ = 0.52) and BY (R^2^ = 0.44) have shown a positive effect on seed yield, indicating that biomass and crop duration contributed to the yield advantage under stress. During the grain filling stage, both the normalized difference vegetation index (NDVI) and chlorophyll (Chl) exhibited consistently positive impacts on grain yield under both HSE and EHSE conditions. This could be attributed to the enhanced photosynthesis resulting from delayed senescence and improved assimilate remobilization under terminal heat stress. The biochemical activity of superoxide dismutase (SOD), peroxidase (POX), and ascorbate peroxidase (APX) was induced in tolerant genotypes under HSE. The correlation of canopy temperature with phenological-physio-biochemical traits remained static under HSE and EHSE, suggesting CT as the best selection parameter for heat tolerance. The traits showing a positive association with yield and that are less affected under stress could be used for selecting tolerant genotypes under stress environments. These tolerant genotypes can be used to develop mapping populations to decipher the genes conferring tolerance as well as to study the molecular basis of tolerance.

## Introduction

As the world population is growing at an exponential rate, meeting the growing demand for wheat products necessitates more concentrated efforts to improve the production and productivity of wheat in a changing climate scenario. As per the global climate models, the mean ambient temperature is predicted to increase by 1.5°C within the next two decades (Masson-Delmotte et al., 2021), and the crop plants will be exposed to heat stress. Wheat is one of the most important cereal crops cultivated throughout the world in terms of area planted and makes a significant contribution to global cereal production (28%) and trade (41.5%) (FAO, 2020). Despite being the world’s second-largest producer of wheat, India’s average productivity is only 2770 kg per hectare, compared to China’s 3885 kg and the UK, the Netherlands, and other NW European nations’ 8043 kg. Higher temperatures, especially during grain filling, shorter crop duration, and a shorter grain filling period are to blame for India’s decreased yield (Mitra and Bhatia, 2008). Continual heat stress (average daily temperature over 17.5°C in the season’s coldest month) affects approximately 7 million ha of wheat in developing countries, while terminal heat stress is a problem in 40% of temperate environments, which cover 36 million hectares (Reynolds et al., 2010). The wheat crop is crucial to the food security of India, and there is a continuous need to enhance the productivity of wheat to meet the food requirements of a fast-growing population (Singh et al., 2012). It has been shown that the 0.4°C increase in global temperature between 1980 and 2000 had a negative effect on wheat productivity (Lobell and Field, 2007). At present, most cereal breeding programs generally deal with limited variability or germplasm to breed new wheat varieties, leading to narrow genetic diversity. This necessitates rethinking and redesigning the breeding strategy to channel newer diversity to breed genotypes with higher productivity and better tolerance to stresses posed by changing climate. The evaluation and identification of germplasm at hot spot locations or by creating artificial stress environments is required. Also, the identification of key traits that contribute more towards tolerance could be useful in selecting desirable genotypes. In India, heat stress is affecting the wheat crop more than other abiotic stresses to which it is exposed (Joshi et al., 2007). Like other C3 species, wheat (*Triticum aestivum* L.) is not physiologically suited to thrive in hot environments, particularly at the grain filling stage. According to Lawlor and Mitchell (2000), an increase of 1°C in temperature during grain filling reduces the harvest index and grain yield of wheat by a proportionate amount and shortens this period by 5%. The impacts of heat stress on reducing photosynthesis, affecting respiration, inactivating enzymes, and rupturing membranes can significantly reduce biomass production (Djanaguiraman et al., 2018; Djanaguiraman et al., 2020). In the Southern Plains, where high temperatures are frequent during crucial developmental stages, the negative effects of heat stress on plant productivity are particularly obvious in winter wheat during seedling establishment (late summer) and grain-filling stages (late spring) (Singh et al., 2012). However, heat stress-induced severity depends on the duration and intensity of stress as well as on genotypes (Kumar et al., 2022). Therefore, it is important to focus on maintaining wheat yields by identifying genotypes that are tolerant to heat stress and promoting breeding techniques and management methods that can protect the wheat output from heat stress (Posch et al., 2019). Practical ramifications of a better understanding of the morpho-physio-biochemical characteristics linked to heat stress tolerance include the identification of different tolerance mechanisms and their application in alleviation treatments (Langridge and Reynolds, 2021; Rehman et al., 2021). Wardlaw and Wrigley (1994) discovered that yield reductions are linked to two types of temperature stress in wheat. The first type is chronic heat stress, characterized by high temperatures throughout the growth cycle, with a mean temperature ranging from 18°C to 25°C and maximum daytime temperatures reaching up to 32°C during grain filling. The second type is heat shock, which occurs when temperatures exceeding 32°C occur during the mid- or late-reproductive stages of wheat, including grain filling. In a study conducted by Wardlaw et al. (2002), contrasting effects of chronic heat stress and heat shock on kernel weight and flour quality in wheat were observed. Heat stress during anthesis results in decreased pollen viability and seed set (Saini and Westgate, 1999; Dolferus et al., 2011; Khan et al., 2022). When stress occurs during grain filling, it leads to diminished starch and protein accumulation (Bhullar and Jenner, 1985; Zahedi et al., 2003), hastened plant development, premature leaf senescence, and reduced photosynthetic rate and capacity (Stone and Nicolas, 1995; Tewolde et al., 2006). Ultimately, these effects culminate in smaller grain size (Wardlaw and Wrigley, 1994; Stone and Nicolas, 1995; Sharma et al., 2008; Talukder et al., 2014) and lower grain yield (Tewolde et al., 2006; Talukder et al., 2014; Kumar et al., 2021; Kumar et al., 2023a). Plants employ various major adaptive mechanisms to develop thermotolerance. These mechanisms involve the increased production of thermo-protectants like secondary metabolites, compatible solutes, ROS scavenging mechanisms, and heat shock proteins (HSPs) (Nakamoto and Hiyama, 1999; Sakamoto and Murata, 2002; Wahid et al., 2007; Mittler et al., 2012). Under severe heat stress, the generation of reactive oxygen species (ROS) as a by-product of aerobic metabolism can have detrimental effects on cellular metabolism, such as lipid membrane peroxidation and damage to nucleic acids and proteins (Bita and Gerats, 2013). To counteract this, plants activate enzymatic and non-enzymatic ROS scavenging systems. The primary ROS-scavenging enzymes include superoxide dismutase (SOD), catalase (CAT), peroxidase (POX), ascorbate peroxidase (APX), and glutathione reductase (GR), while non-enzymatic systems involve ascorbic acid (ASC) and glutathione (GSH) (Suzuki et al., 2012). Maintaining elevated levels of these antioxidants is crucial for enhancing thermotolerance in plants (Awasthi et al., 2014). However, owing to the complex nature of thermotolerance, it demands a multidisciplinary, holistic approach integrating the outcomes of biochemical, physiological, breeding, and agronomic interventions to sustain wheat production under current and future changing climates (Yadav et al., 2022). In the literature, most of the heat tolerance studies in wheat are undertaken on large germplasm, and that too focused either on morphological or physiological traits. Enhanced activity of biochemical traits like superoxide dismutase (SOD), peroxidase (POX), and ascorbate peroxidase (APX) is also important in providing tolerance under stress environments. Therefore, the present study was undertaken to (1) assess the differences in the performance of tolerant and heat sensitive genotypes under heat and extended heat stress on yield-contributing biochemical and physiological traits; (2) to identify key traits that could be useful in selecting and breeding wheat for heat tolerance.

## Materials and methods

The present investigation was conducted at the Experimental Research Farm of the Department of Genetics and Plant Breeding, BUAT, Banda (25.5269°N Latitude and 80.3418°E Longitude). The present investigation was undertaken with the objective of studying the effects of short- to long-duration terminal heat stress on wheat by exposing plants to heat stress conditions through delayed sowing.

### Plant material

The experimental material was identified based on the phenotypic screening of 123 bread wheat cultivars under open field conditions during rabi season 2020-21 at BUAT-Banda. Out of 123 cultivars, eight contrasting wheat cultivars viz., four heat tolerant (DBW 150, WH 730, AKAW 2862-1, and Halna) and four heat sensitive (K 1006, PBW 550, WH 1105, and HD 2967) cultivars were identified based on Heat Tolerance Index (HTI), Heat Susceptibility Index (HSI), Canopy Temperature (CT), Capacity for Grain Filling (KWR %) and Yield Stability Index (YSI). All of these genotypes were of the spring type and were collected from ICAR- Indian Institute of Wheat and Barley Research, Karnal (India). These eight contrasting cultivars were evaluated to study the effect of heat stress on different phenological, physiological, and biochemical traits in bread wheat during crop season 2021-22.

### Field growing conditions

**Figure 1:**
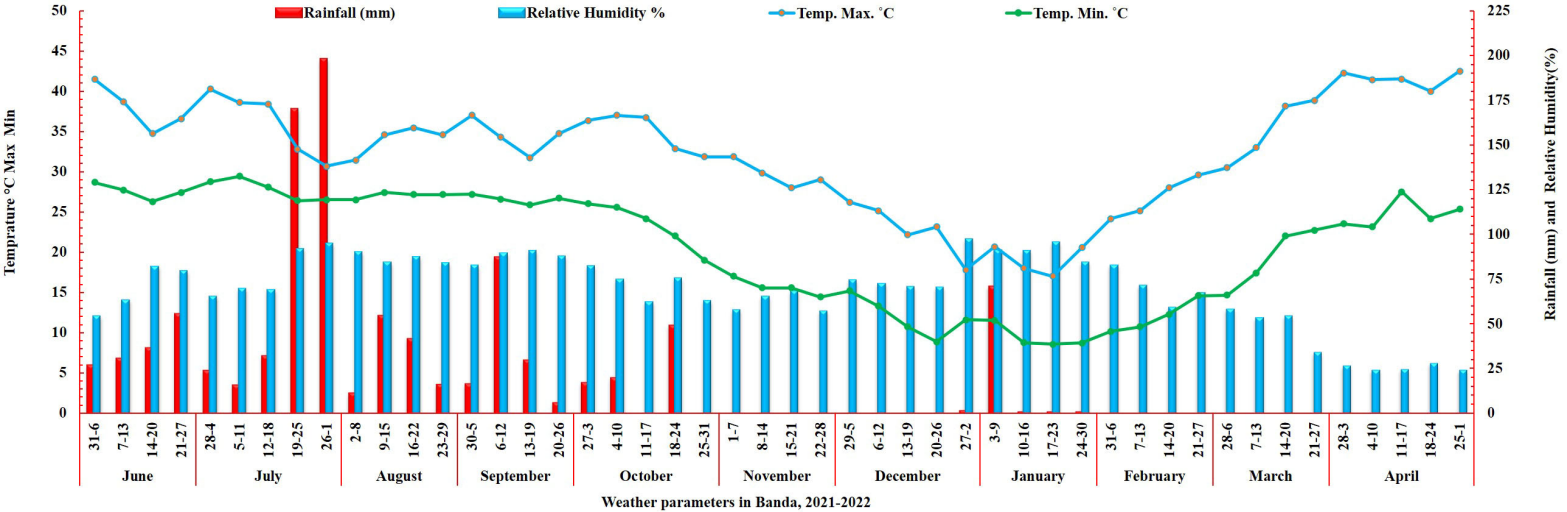
Weather data on rainfall (mm), humidity, and temperature during the 2021-22 crop season.

The weather in Banda is characterized by high temperatures, frequent droughts, and unpredictable precipitation, with an average annual rainfall of 850 mm. The climatic conditions in this region make it an ideal location for conducting terminal heat stress screening for cool-season crops, such as wheat. The data on weather parameters was obtained from the Meteorological Observatory Unit (MOU) at the BUAT campus. The annual rainfall (mm), maximum, minimum temperature, and relative humidity during the entire crop growth period are presented in **Figure 1**.

The experimental material was planted in a two-row plot measuring 2 m in length, with two replications following a randomized complete block design. The distance between plants and rows was maintained at 10 cm and 25 cm, allowing for approximately 40 plants in each plot (per genotype). Experiments were conducted in three different environments, as described below:

I. Optimum environment (OE): The crop was sown in the first week of November.
II. Heat stress environment (HSE): The crop was sown in the second week of December.
III. Extended heat stress environment (EHSE): The crop was sown in the first week of January.

Both the OE and HSE experiments were irrigated five times to avoid the effect of drought on the test material. The EHSE experiment was irrigated every 8-10 days to avoid the confounding effects of heat and drought stress. The recommended practice packages for this region in terms of fertilization, weeding, and crop protection were adopted to grow healthy wheat crops.

### Data collection

A total of 21 traits such as phenological, physiological, and biochemical traits were recorded in each contrasting cultivar, as mentioned below.

### Phenological traits

Data on 10 important phenological traits were recorded in all three environments. The traits included 50% flowering (FLW), days to maturity (DM), plant height (PH in cm), number of tillers per plant (NT), spike length (SL in cm), spikelet/spike (SS), grain number/spike (GS), test kernel weight (TKW in g), biological yield (BY), and seed yield (SY). The FLW and DM were recorded on a plot basis when 50% of the plants reached heading and when the whole plot crop reached physiological maturity. PH, NT, SL, SS, and GS were measured on five randomly selected plants, and average values were taken for further analysis. The BY and SY were recorded in kg per plot for each genotype, while the TKW was estimated by counting 100 seeds in grams (g).

### Physiological traits

Data on four relevant physiological traits, *viz*., canopy temperature (CT), normalized difference vegetation index (NDVI), chlorophyll content index (CCI), and chlorophyll concentration in µmol m^2^ (Chl), were recorded at the heading and grain filling stages of the crop (Pask et al., 2012).

### Canopy temperature °C

The canopy temperature of the plot was measured using a portable infrared radiometer (Apogee, MI-210) at the heading and mid-grain filling stages. The readings were recorded between 11:00 and 14:00, when plants were exposed to the highest temperatures. The five CT readings from each plot were recorded, and the mean values were used for analysis.

### Normalized difference vegetation index

The NDVI was measured using a portable infrared radiometer (Apogee, AT-100) at the heading and grain filling stages for each plot. The readings were measured under bright, sunny, and clear skies. The five observations from each plot were recorded for 20 seconds and the mean values were used for analysis.

### Chlorophyll content

The chlorophyll content index (CCI) and the chlorophyll concentration in µmol m^2^ (Chl) of the middle part of the flag leaf were measured using a chlorophyll concentration meter (Apogee, MC-100) at the mid grain-filling stage. The three readings were taken from plant leaves, and five plants were selected from one plot and connected directly to the data logger. The average values of each of the 15 samples were used for data analysis.

### Biochemical parameters

Leaf samples were collected at 15 DAA (days after anthesis) and subjected to biochemical studies.

### Extraction and estimation of antioxidant enzymes

All enzymes were extracted in triplicate (replication-wise) with appropriate extraction buffers at 4°C and assayed at 37°C. Glutathione reductase (GR) and SOD were extracted by homogenizing the samples in 0.1 M phosphate buffer (pH 7.5) containing 1% polyvinylpyrrolidone (PVP), 1 mM EDTA, and 10 mM β-mercaptoethanol using a pre-chilled pestle and mortar. CAT and APX were extracted with 0.05 M phosphate buffer (pH 7.5) containing 1% PVP (Kaur et al., 2008). After centrifugation of the homogenates at 10,000*g* for 20 min, the supernatant was used for the assay.

The activity of SOD was determined by adding 100 mM Tris HCl buffer (pH 8.2), 6 mM EDTA, and 6 mM pyrogallol solution to the enzyme extract as described by Marklund and Marklund (1974), with partial modifications in the concentrations of the components (Singh et al., 2023). The change in absorbance was recorded at 420 nm after an interval of 30 sec up to 3 min. One unit of enzyme activity was defined as the amount of enzyme that caused 50% inhibition of the auto-oxidation of pyrogallol observed in the blank in one minute in one gram of fresh tissue.

GR activity was assayed by adding 200 mM potassium phosphate buffer (pH 7.5), 1.5 mM MgCl_2_, 0.2 mM EDTA, and 0.025 mM NADPH to the enzyme extract, followed by 0.25 mM oxidized glutathione in the reaction mixture (Esterbauer and Grill, 1978). Partial modifications of the original method included the use of phosphate buffer (0.2M, pH 7.5) instead of Tris-HCl (0.1 M, pH 7.4) and modified concentrations of the other assay components to achieve better results (Kaur et al., 2023). The decrease in absorbance at 340 nm was recorded after an interval of 30 sec up to 3 min. One unit of GR activity was expressed as nmoles of NADP^+^ formed min^-1^g^-1^ of FW and was calculated using €_NADPH_ of 6.22 mM^-1^ cm^-1^.

The assay mixture of APX included 50 mM sodium phosphate buffer (pH 7.0), 0.5 mM ascorbic acid in enzyme extract, and H_2_O_2_ solution (Nakano and Asada, 1987). Absorbance was recorded at 290 nm in a spectrophotometer after an interval of 30 sec up to 3 min. The molar extinction coefficient of monodehydroascorbic acid (MDAA) was 2.8 mM^-1^cm^-1^ and the enzyme activity was expressed as nmoles of MDAA formed min^-1^g^-1^ of FW.

CAT activity was determined by preparing a reaction mixture containing 50 mM sodium phosphate buffer (pH 7.5) and enzyme extract. The reaction was initiated by the addition of H_2_O_2_ solution: its consumption was recorded at 240 nm for 3 min at 30-sec intervals (Chance and Maehly, 1955). The extinction coefficient for H_2_O_2_ was 0.0394 mM^-1^cm^-1^. Enzyme activity was expressed as μmoles of H_2_O_2_ decomposed min^-1^g^-1^ of FW.

### Extraction and estimation of proline

The method by Bates et al. (1973) was used to measure the amount of proline. Extraction was performed in a sulfosalicylic acid solution (3% w/v), followed by centrifugation at 6000 g for 15 min. The assay mixture included an acid-ninhydrin solution and glacial acetic acid in addition to the extract. The reaction mixture was boiled at 100°C for 1 h, followed by the addition of toluene. The toluene-containing chromophore was aspirated from the aqueous phase, and the absorbance was read at 520 nm and measured in a spectrophotometer. Proline content was calculated using the standard curve and expressed as mg g^−1^ FW.

### Statistical analysis

The combined analysis of variance for all pheno-physiological and biochemical traits was computed using the STAR, I (2014). The genotypes, treatments, their interactions, standard errors, and least significant difference (LSD) test along with Tukey’s Honest Significant Difference (HSD) test, were performed. Correlation analysis was performed for each environment and combined using the corr_plot function of the “metan” package (Olivoto and Lúcio, 2020) in R software. The linear regression analysis and scatter plot of SY taken as a dependent variable with all traits considered independent variables were performed using tidyverse, coherent rstatix (Wickham et al., 2019), and ggplot2 (Wickham, 2016) packages in R software.

## Results

The analysis of variance showed a significant effect of genotypes, environments, and genotype x environment interaction, indicating that the expression of genotypes changed in different environments for all the measured traits (**Table 1**). The comparative analysis of cultivar performance under different environments demonstrated a high impact of heat stress on all the traits studied.

**Table 1:** Analysis of variance for phenological-physio-biochemical traits in contrasting heat-tolerant and heat-susceptible wheat cultivars used in the study.

S. No.	Source of variation\ Df	Environment	Replication within Env	Cultivar	Environment: Cultivar
		2	3	7	14
Phenological					
1	FLW	2158.39***	0.18	194.75***	14.41***
2	DM	9801.18***	0.10	115.25***	16.47***
3	PH	9801.18***	0.10	115.25***	16.47***
4	NT	127.02***	4.60***	10.02***	6.37***
5	SL	12.84***	0.36	8.84***	0.92*
6	SS	51.39***	0.68	16.90***	6.71***
7	GS	1322.17***	4.58	147.54***	83.24***
8	TKW	4.11*	0.15***	0.34***	0.28***
9	BY	4.64***	0.023	0.29***	0.16***
10	SY	1.94***	0.03	0.04***	0.028***
Physiological					
11	CT_HS_	687.55***	0.010	14.53***	4.48***
12	CT_GFS_	1227.44***	0.25	5.19***	29.57***
13	NDVI_HS_	0.13***	0.001	0.04***	0.02***
14	NDVI_GFS_	0.04***	0.02	0.04***	0.02***
15	CCI_GFS_	120.13***	0.89	60.92***	20.04***
16	Chl_GFS_	5252.74***	258.90	10596.15***	6803.02***
Biochemical					
17	SOD	4404.99***	3.65	1692.40***	316.7***
18	POX	358.26***	2.35***	122.38***	25.02***
19	APX	1987843.25***	13097.34***	360732.96***	152110.20***
20	CAT	110715.66***	308.26***	2366.50***	6106.05***
21	Proline	0.04***	0.0080***	0.02***	0.02***

* Significant at a probability of .05.

** Significant at a probability of .01.

*** Significant at a probability of .001.

FLW, Days to 50% flowering; DM, Days to maturity; PH, Plant height (cm); NT, Number of tillers per plant; SL, Spike length (cm); SS, Number of spikelets/spike; GS, Grain number/spike; TKW, Test kernel weight (gm); BY, Biological yield (kg/plot); SY, Seed yield (kg/plot); CT_HS,_ Canopy temperature at heading stage; CT_GFS,_ Canopy temperature at mid gain-filling stage; NDVI_HS,_ Normalized difference vegetation index at the heading stage; NDVI_GFS,_ Normalized difference vegetation index at the mid gain-filling stage; CCI_GFS,_ Chlorophyll concentration index (CCI) at the mid gain-filling stage; Chl_GFS,_ Chlorophyll concentration (µmol m2) at the mid gain-filling stage; SOD, Superoxide dismutase; POX, Peroxidase; APX, Ascorbate peroxidase; CAT, Catalase activity; Proline, Proline content.

### Phenological traits

**Figure 2:**
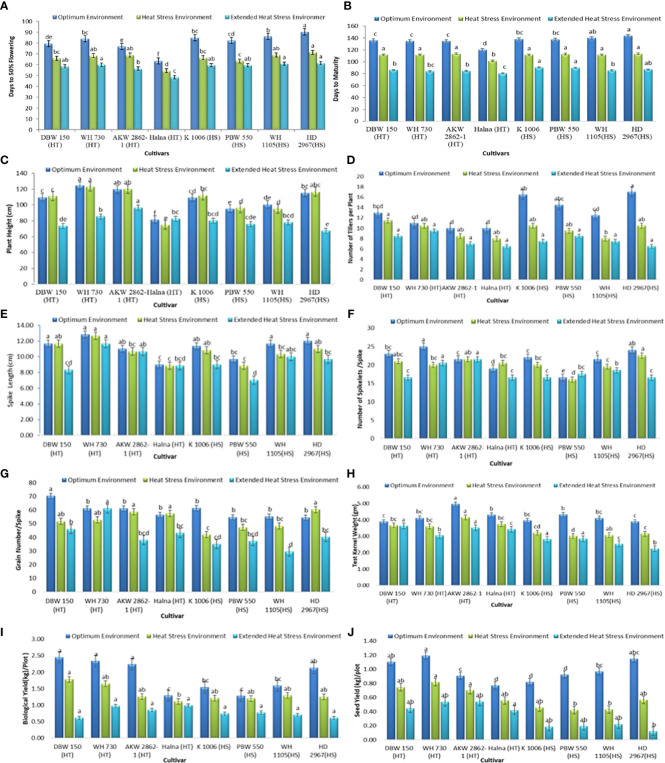
Phenological traits **(A)** FLW **(B)** DM **(C)** PH **(D)** NT **(E)** SL **(F)** SS **(G)** GS **(H)** TKW **(I)** BY and **(J)** SY under optimum environment, heat stress environment (HSE) and extended heat stress environment (EHSE). Letters a, b, c, d and e on bar indicates significant differences between variety-temperature combinations based on Tukey’s HSD test. Means with the same letter are non-significant.

There were significant effects of a gradual increase in temperature observed in each genotype for all phenological traits (**Figure 2**).

i. Days to 50% flowering (FLW): The average FLW under OE was 81 days, 66 days in HSE, and 58 days in EHSE (**Figure 2A**, **Table 2**). The FLW ranged from 63 to 91 days in OE, 54-72 days in HSE, and 48-62 days in EHSE. Halna was the earliest parental line among 64 days in OE, 55 days in HSE, and 49 days in EHSE. The HD 2967 was the last and took the maximum amount of time to head into each environment. The FLW showed an 18% reduction in HSE and a 28% reduction in time in EHSE as compared to the FLW in OE (**Supplementary Material, Table A1**).

**Table 2:** Descriptive statistics of 21 phenological, physiological, and biochemical traits recorded in the contrasting varieties under optimum, heat stress and extended heat stress environments.

Trait	Optimum Environment				Heat Stress Environment				Extended Heat Stress Environment			
	Range	Mean	SD	CV	Range	Mean	SD	CV	Range	Mean	SD	CV
Phenological Traits												
FLW	63-91	81 ± 1.98	7.93	9.79	54-72	66.06 ± 1.28	5.12	7.75	48-62	58.13 ± 1.01	4.06	6.99
DM	119-144	135.5 ± 1.69	6.77	5.00	101-114	110.94 ± 0.96	3.82	3.44	80-91	86 ± 0.80	3.20	3.73
PH	80-126	106.81 ± 3.40	13.61	12.74	70-123	106.06 ± 3.95	15.80	14.89	66-98	79.94 ± 2.15	8.58	10.74
NT	9-19	13.06 ± 0.76	3.02	23.13	7-12	9.5 ± 0.40	1.59	16.75	6-10	7.5 ± 0.32	1.26	16.87
SL	9-13.67	11.15 ± 0.34	1.36	12.21	8.67-13	10.60 ± 0.32	1.29	12.15	7-12	9.40 ± 0.35	1.41	15.06
SS	16-25	21.56 ± 0.67	2.68	12.44	16-23	20.13 ± 0.47	1.89	9.41	16-22	18 ± 0.5	2.00	11.11
GS	51-73.50	59.31 ± 1.43	5.74	9.67	41-60.5	52.31 ± 1.54	6.16	11.77	25-62	41.28 ± 2.32	9.29	22.50
TKW	3.88-4.97	4.19 ± 0.09	0.34	8.18	3.01-4.17	3.62 ± 0.08	0.33	9.09	2.22-3.93	3.18 ± 0.13	0.50	15.78
BY	1.18-2.75	1.85 ± 0.13	0.51	27.59	1.06-1.81	1.34 ± 0.06	0.24	18.07	0.60-1.00	0.77 ± 0.03	0.14	18.45
SY	0.64-1.25	0.91 ± 0.05	0.20	21.63	0.42-0.63	0.51 ± 0.02	0.08	15.40	0.12-0.32	0.22 ± 0.01	0.06	25.34
Physiological traits												
CT_HS_	19.13-24.27	21.82 ± 0.46	1.82	8.36	17.67-22.97	20.14 ± 0.41	1.65	8.18	29.40-36.73	32.24 ± 0.56	2.26	7.00
CT_GFS_	19.37-25.60	23.33 ± 0.51	2.06	8.82	28.67-34.87	31.86 ± 0.52	2.09	6.56	33.83-46.63	40.85 ± 1.169	4.68	11.45
NDVI_HS_	0.53-0.69	0.62 ± 0.011	0.04	7.10	0.61-0.69	0.573 ± 0.01	0.02	3.24	0.42-0.53	0.46 ± 0.01	0.03	7.04
NDVI_GFS_	0.42-0.59	0.60 ± 0.01	0.05	9.46	0.51-0.58	0.49 ± 0.01	0.02	3.80	0.31-0.59	0.37 ± 0.02	0.09	20.35
CCI_GFS_	19.60-37	31.54 ± 1.09	4.37	16.45	26.30-37	29.37 ± 0.92	3.69	11.76	24.30-39	28.2 ± 0.99	3.99	12.79
Chl_GFS_	415.10-611.8	556.81 ± 14.19	56.77	10.44	416.60-601.50	483.22 ± 13.47	53.92	10.61	377.60-679.30	411.16 ± 19.59	78.36	15.07
Biological Traits												
SOD	75.5-111.9	88.27 ± 2.48	9.93	11.26	59.27-146.88	92.71 ± 7.10	28.41	30.65	40.58-80	62.01 ± 3.40	13.58	21.91
POX	18.88-28.28	22.31 ± 0.62	2.50	11.21	14.82-37.3	24.25 ± 1.96	7.83	32.31	10.15-20.62	15.25 ± 0.92	3.70	24.24
APX	732.35-922.36	805.78 ± 13.76	55.04	6.83	589.62-2342.40	1273.03 ± 127.22	508.88	39.97	323.36-1100.5	582.27 ± 56.48	225.94	38.80
CAT	307.56-368.94	330.13 ± 4.79	19.15	5.80	143.44-344.34	244.89 ± 18.08	72.32	29.53	110.60-244.91	163.77 ± 8.91	35.64	21.76
Proline	0.08-0.11	0.09 ± 0.002	0.01	11.30	0.09-0.15	0.11 ± 0.01	0.02	21.33	0.06-0.13	0.09 ± 0.01	0.02	24.411

FLW, Days to 50% flowering; DM, Days to maturity; PH, Plant height (cm); NT, Number of tillers per plant; SL, Spike length (cm); SS, Number of spikelets/spike; GS, Grain number/spike; TKW, Test kernel weight (gm); BY, Biological yield (kg/plot); SY, Seed yield (kg/plot); CT_HS,_ Canopy temperature at heading stage; CT_GFS,_ Canopy temperature at mid gain-filling stage; NDVI_HS,_ Normalized difference vegetation index at the heading stage; NDVI_GFS,_ Normalized difference vegetation index at the mid gain-filling stage; CCI_GFS,_ Chlorophyll concentration index (CCI) at the mid gain-filling stage; Chl_GFS,_ Chlorophyll concentration (µmol m2) at the mid gain-filling stage; SOD, Superoxide dismutase; POX, Peroxidase; APX, Ascorbate peroxidase; CAT, Catalase activity; Proline, Proline content.

ii. Days to maturity (DM): The duration of crop maturity is an important factor to avoiding terminal heat stress. The average DM in the heat-tolerant and heat-susceptible parents was 131 and 139 days, respectively, under optimum environment (OE) (**Table 2**). The average maturity duration was reduced to about 25 days (18%) under HSE and EHSE (36%) in heat-tolerant and heat-susceptible parents (**Figure 2B**; **Supplementary Material, Table A2**). The crop cycle was drastically shortened, leading to forced maturity in EHSE as compared to OE. Parent HD2967 took the maximum amount of time to reach maturity and was of longer duration in all environments.

iii. Plant Height (HT): Plant height was significantly reduced, to about 36% under EHSE as compared to OE. Plant height showed less reduction (4%) under HSE as compared to OE (**Figure 2C**; **Supplementary Material, Table A3**). Halna was the shortest line and remained unaffected in all conditions, and WH730 was the tallest genotype in all environments.

iv. Number of Tillers/Plant (NT): TN ranged from 9-19 under OE, 7-12 under HSE, and 6-10 under EHSE. The average number of tillers per plant was high in heat-susceptible parents (15) as compared to heat-tolerant parents (11) under OE (**Figure 2D**). The number of tillers per plant showed a reduction of 39% under EHSE (**Supplementary Material, Table A4**). A maximum reduction of 38% and 62% was observed in HD 2967 under HSE and EHSE, respectively.

v. Spike length (SL): Spike length ranged from 9.0-13.67 under OE, 8.67-13.0 under HSE, and 7-12 under EHSE (**Figure 2E**; **Table 2**). Spike length was drastically reduced under EHSE, and the maximum reduction (15.56%) was observed under EHSE. Spike length was more affected in HD2967.

vi. Number of spikelets/spike (SS): The number of spikelets/spike was not significantly affected by temperature stress. The average number of spikelets per spike was 21 in OE, 20 in HSE, and 18 in EHSE (**Figure 2F**; **Table 2**). The maximum reduction of 20% in SS was recorded in EHSE.

vii. Number of grains per spike (GS): The average GS recorded a reduction of 32% and 14% under EHSE and HSE, respectively, in comparison to OE (**Figure 2G**; **Supplementary Material**, **Table A7**). The highest reduction in GS (47%) was observed in WH 1105, while the minimum reduction (16%) was observed in WH 730 under EHSE (**Figure 2**).

viii. Test kernel weight (TKW): The average 100 seed weight was reduced by 17.54% and 27.33% under HSE and EHSE, respectively, compared to OE. The maximum shriveled grain (42.31%) was recorded in cultivar HD 2967, while the minimum reduction (6.42%) was observed in DBW 150 (**Figure 2H**, **Supplementary Material, Table A8**).

ix. Biological yield (BY): Biological yield was also severely affected by heat stress under HSE and EHSE. The average BY (1.85 kg/plot) was recorded under OE, 1.34 kg/plot under HSE, and 0.78kg/plot under EHSE. The BY showed a reduction of 17% in HSE and 42% in EHSE in heat-tolerant parents, whereas a reduction of 23% and 54% was observed under HSE and EHSE in heat-susceptible parents, respectively (**Figure 2I**; **Supplementary Material, Table A9**).

x. Seed Yield (SY): The heat-tolerant parental lines showed the highest yield reduction under HSE (28.57%) and EHSE (50.0%), whereas a yield reduction of 51% and 81% was observed under HSE and EHSE environments in heat-susceptible parents (**Figure 2J**; **Supplementary Material, Table A10**). HD 2967 (89% reduction) was highly affected by prolonged heat stress, showing yield in EHSE.

### Physiological traits

**Figure 3:**
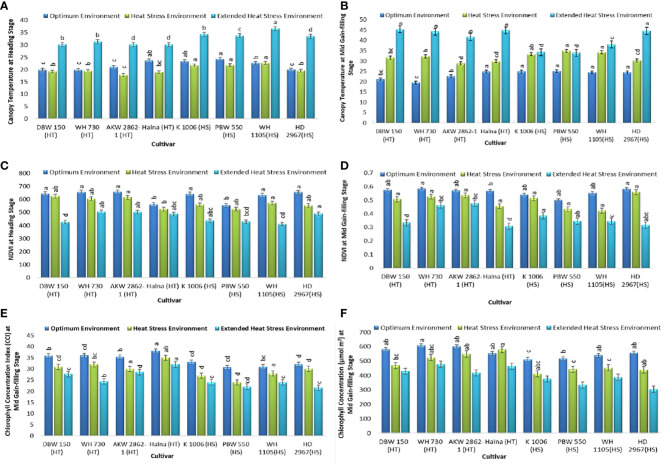
Physiological traits **(A)** CT_HS_ **(B)** CT_GFS_, **(C)** NDVI_HS_ **(D)** NDVI_GFS_ **(E)** CCI_GFS,_ and **(F)** Chl_GFS_, under optimum environment, heat stress environment (HSE) and extended heat stress environment (EHSE). Letters a, b, c and d on bar indicates significant differences between variety-temperature combinations based on Tukey’s HSD test. Means with the same letter are non-significant.

The canopy temperature (CT) at the heading stage was <35°C, which does not affect the reproductive ability of the plant. The average CT recorded at the heading stage was 21.82°C and 20.14°C in OE and HSE, respectively. The CT at the heading stage was 32.24°C in the very late-sown experiment. At the grain filling stage, the average CT was 23.33°C in OE and 31.86°C in HSE, and a high temperature of 40.85°C was recorded in EHSE at the mid-grain filling stage of the crop. Thus, the late-sown crop was exposed to extensive heat stress under the prolonged heat stress environment of EHSE (**Figures 3A, B**). The NDVI (Normalized Difference Vegetation Index) is a simple graphical indicator that is used to analyze the sample based on the difference in measurement of near-infrared and red light. The NDVI was high at the heading stage (622 measured in OE, 573 measured in HSE, and 461 measured in EHSE), whereas the NDVI values showed a reduction at the grain filling stage (600 in OE, 490 in HSE, and 370 in EHSE). Plant greenness was significantly reduced in the heat-stress environment as compared to the optimum environment (**Figures 3C, D**). The CCI was higher in OE as compared to the heat-stress environment at the grain filling stage. The average chlorophyll concentration was 556 μmol m^2^ in OE, 483 μmol m^2^ in HSE, and 411 μmol m^2^ in EHSE (**Figures 3E, F**).

### Biochemical traits

**Figure 4:**
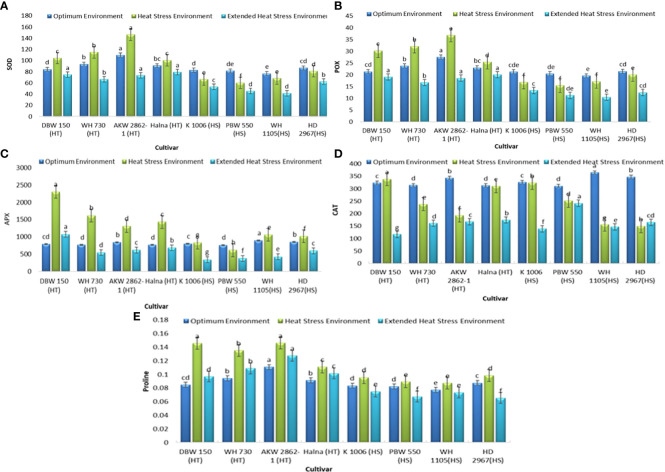
Biochemical traits **(A)** SOD (units min^-1^gm ^-1^ of FW), **(B)** POX (ΔA min^-1^gm^-1^ of FW), **(C)** APX (nmoles of MDA formed min^-1^gm^-1^ FW), **(D)** CAT (µmoles of H_2_O_2_ decomposed min^-1^g^-1^ of FW), and **(E)** Proline content (mg/gm FW) under optimum environment, heat stress environment (HSE) and extended heat stress environment (EHSE). Letters a, b, c and d on bar indicates significant differences between variety-temperature combinations based on Tukey’s HSD test. Means with the same letter are non-significant.

The biochemical studies related to understanding the mechanism of heat tolerance were also undertaken with a major focus on the antioxidant system (**Figure 4**). It was observed that the activity of SOD was moderately to significantly induce in all the tolerant genotypes under heat stress as compared to the control samples, with a maximum increase of 33% recorded in AKW2862-1, while all the susceptible genotypes showed a decline in activity in heat-stressed samples, with the highest decrease of 26% observed in PBW 550. All heat-tolerant and heat-susceptible genotypes showed a decrease in SOD activity under extended heat stress conditions when compared with controls. The trend for POX activity was also found to be similar to that of SOD. The rise in POX activity in tolerant genotypes ranged from 10.4% (Halna) to 41% (DBW 150). Like SOD, POX activity in susceptible genotypes also showed a decreasing trend under stress conditions, with the highest decrease of 24% recorded for PBW 550 under heat stress, while other susceptible genotypes also showed moderate to slight decreases in activity. Both sets of genotypes showed decreased POX activity under extended heat stress; however, this reduction was more pronounced in heat-susceptible genotypes as compared to heat-tolerant ones.

APX activity was significantly increased in tolerant genotypes under heat stress compared to controls. The increase in activity ranged from 193% (DBW 150) to 56.6% (AKAW 2862-1) when compared to control samples, whereas only a slight increase in activity was observed in the case of all the susceptible genotypes. A decrease in APX activity under extended heat stress was observed in both genotypes except in DBW 150, where the activity was 37% higher than the control samples, although the activity was 53% lower than the activity under heat stress. CAT activity was found to either remain unchanged (DBW 150 and Halna) or significantly decreased (WH 730 and AKW 2862-1) in tolerant genotypes, while the activity was significantly decreased in all the susceptible genotypes except for K 1006, in which the activity remained unchanged. All genotypes showed a decline in APX activity under extended heat stress as compared to the activity recorded under optimum and heat stress conditions.

The data presented in **Table 2** and **Figure 4** showed that both the tolerant and susceptible genotypes accumulated higher proline content under heat stress conditions as compared to the control samples. The highest increase of 72% in proline content was recorded for DBW 150. followed by WH 730, which showed an increase of 44% in proline content as compared to control samples. However, variation in the pattern could be observed under prolonged heat stress in terms of proline synthesis, as tolerant genotypes exhibited a moderate increase in proline content under extended heat conditions, whereas a moderate decrease was observed in heat-susceptible genotypes as compared to the samples analyzed in optimum environments.

Correlation analysis: Correlation heat maps were generated to understand the relationship between phenological, physiological, and biochemical variables in all three individual environments and pooled across environments.

**Figure 5:**
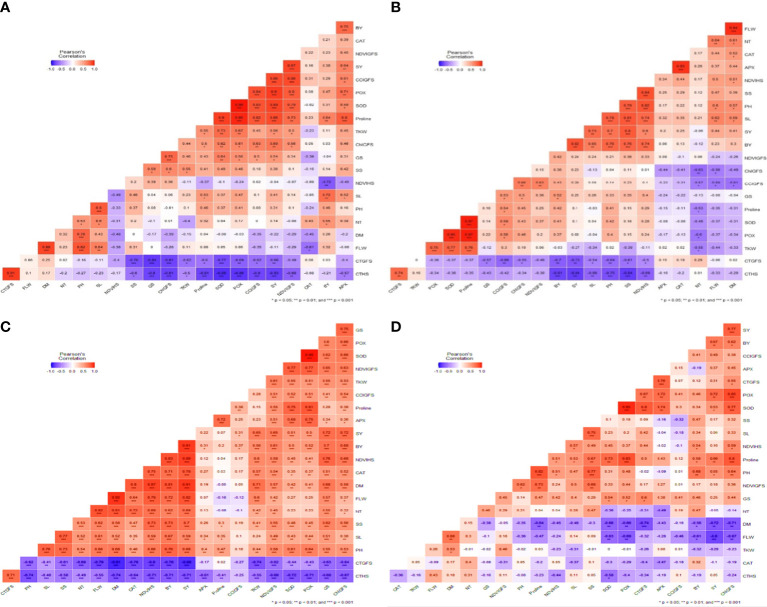
Correlation heat map of all 21 traits under **(A)** Optimum Environment **(B)** Heat Stress Environment (HSE) **(C)**. Extended Heat Stress Environment (EHSE) **(D)** Combined Environment. The blue color indicates a negative correlation, while the red color indicates a positive correlation. The color intensity increases with its significance level.

Optimum environment: SY was significantly and negatively correlated with CT_HS_ (r = - 0.84***) and CT^GFS^ (r = - 0.73**) (**Figure 5A**). The TKW (r=-0.27) and NT (r = -0.06) were not significantly or negatively correlated with SY. SY was significantly and positively correlated with BY (r = 0.82***), SL (r = 0.73**), PH (r = 0.70**), SS (r = 0.80***), and NDVI_HS_ (r = 0.60*), while APX (r =0.20), CAT (r =0.25), FLW (r =0.44), and DM (r =0.41), showed a positive but insignificant correlation. DM was negatively correlated with CT_HS_, CT_GFS,_ TKW, POX, SOD, proline, GS CCI_GFS,_ Chl_GFS,_ and NDVI_GFS,_ while phenological traits had a positive correlation. The CT_HS_ and CT_GFS_ were highly negatively correlated with all the traits studied, while both traits were positively correlated with Chl_GFS and_ TKW.

Heat stress environment: Under a heat stress environment, SY was highly significant and negatively correlated with CT_HS_ (r = -0.83***), and CT_GFS_ (r = -0.66**) (**Figure 5B**), but FLW (r = -0.11), DM (r = -0.22), and NDTV_HS_ (r = -0.04) showed a non-significant negative correlation. SY was significantly and positively correlated with GS (r =0.54*), Chl_GFS_ (r =0.69**), TKW (r =0.56*), proline (r =0.86**), SOD (r =0.89**), POX (r =0.80**), and CCI_GFS_ (r =0.90**).

Extended heat stress environment: Under EHSE, SY was significantly but negatively correlated with the crop cycle traits FLW (r = -0.80***) and DM (r = -0.72**) (**Figure 5C**), but the traits CT_HS_ (r =0.05), CAT (r = - 0.01), TKW (r = - 0.29), and NT (r = - 0.05) showed a non-significant negative correlation with SY. Traits GS and NDVI_GFS_ had a non-significant positive correlation with seed yield. SY under high-temperature stress was significantly and positively correlated with the PH (r =0.66**), Proline (r =0.58**), POD (r =0.80**), SOD (r =0.53*), POX (r =0.72**), and BY (r =0.67**), while SL (r =0.06), SS (r =0.17), CT_GFS_ (r =0.31), APX (r =0.37), and CCI_GFS_ (r =0.49), had a non-significant positive correlation.

Combined correlation: A correlogram (**Figure 5D**) was generated for all three environments, showing the relationship between the parameters. The combined correlation analysis shows that all studied traits are positively correlated with SY except CT_HS_ (r = -0.71**), and CT_HFS_ (r = -0.88**). The correlation between traits and SY changed under different stress environments. For some of the traits, the correlation changed from positive to negative. A few important traits that showed a positive correlation with SY even under EHSE, should be highlighted for the selection of heat-tolerant lines.

**Figure 6:**
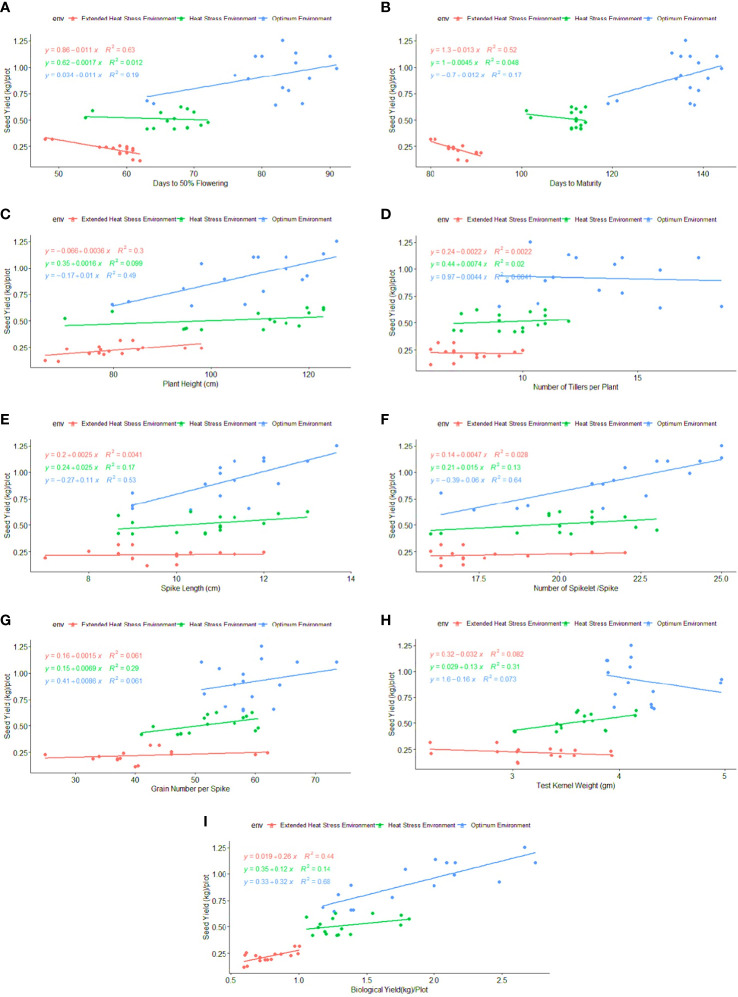
Regression coefficient of phenological traits **(A)** FLW, **(B)** DM, **(C)** PH, **(D)** NT, **(E)** SL, **(F)** SS, **(G)** GS, **(H)** TKW, and **(I)** BY with seed yield (kg/plot) under optimum environment, heat stress environment (HSE) and extended heat stress environment (EHSE).

**Figure 7:**
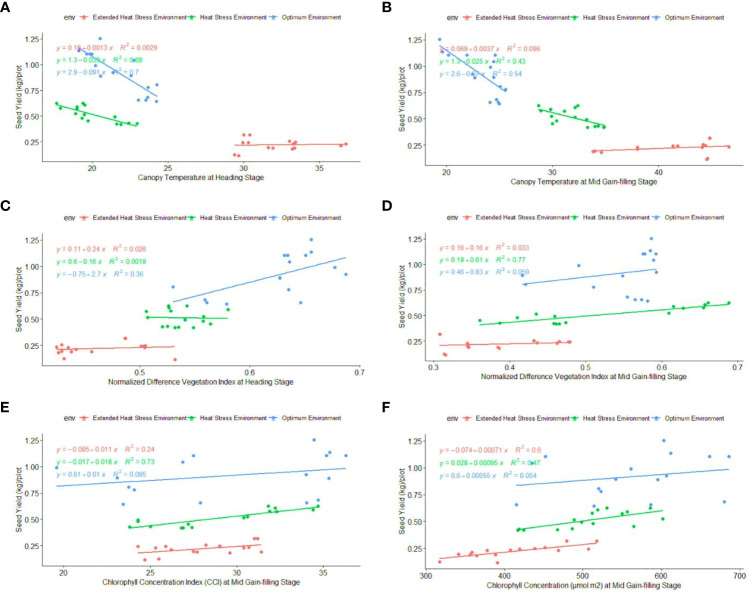
Regression coefficient of physiological traits **(A)** CT_HS_, **(B)** CT_GFS_, **(C)** NDVI_HS_, **(D)** NDVI_GFS_, **(E)** CCI_GFS,_ and **(F)** Chl_GFS_, with seed yield (kg\plot) under optimum environment, Heat stress environment (HSE) and extended heat stress environment (EHSE).

**Figure 8:**
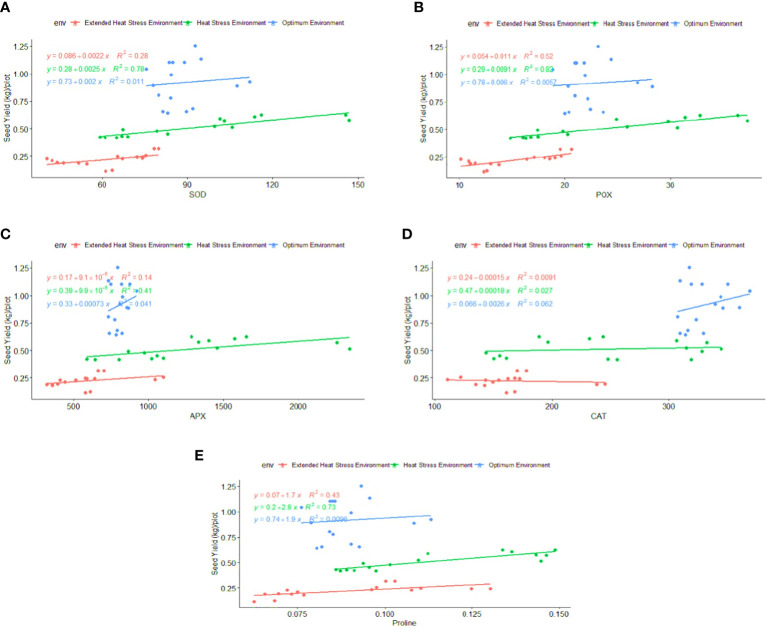
Regression Coefficient of biochemical traits **(A)** SOD **(B)** POX **(C)** APX **(D)** CAT and **(E)** Proline with seed yield (kg\plot) under optimum environment, heat stress environment (HSE) and extended heat stress environment (EHSE).

Regression analysis: A regression analysis was performed to determine the relationship between phenological-physio-biochemical traits and seed yield. Among the phenological traits, DM (R^2 = ^0.52) and BY (R^2 = ^0.44) have shown a positive effect on seed yield, indicating that biomass and crop duration contributed to the yield advantage under stress conditions (**Figure 6**). CT_HS_ and CT_GFS_ had a positive impact on seed yield, with R^2^ = 0.70 and R^2^ = 0.54 under OE and R^2^ = 0.88 and R^2^ = 0.43 under HSE, respectively (**Figure 7**). In EHSE, CT had a non-significant association with seed yield. The NDVI_HS_ showed a significant association (R^2^ = 0.36) with seed yield under OE, while a non-significant association was observed under HSE and EHSE. The NDVI_GFS_ and Chl_GFS_ showed a consistently significant positive effect on grain yield under HSE and EHSE. The strong effect of CCI and Chl_GFS_ on grain yield was observed under HSE, which was found to be reduced under EHSE. Among biochemical traits, SOD, POX, APX, and proline had the maximum positive relationship under HSE and EHSE, while the effect was very low under OE conditions (**Figure 8**).

## Discussion

This study was conducted to assess the effect of moderate to extreme heat stress on phenological-physio-biochemical traits in four heat-tolerant and four heat-sensitive genotypes. Late planting is an effective way to expose plants to terminal heat stress to estimate the effect of high temperature on phenological-physio-biochemical traits (Gupta et al., 2015; Nagar et al., 2015; Sharma et al., 2015; Agarwal et al., 2021; Rehman et al., 2021). The ANOVA analysis indicated significant differences among the genotypes for phenological-physio-biochemical traits under stress conditions, indicating that there are differences among the genotypes with respect to stress tolerance. The reduction in the mean and range for different traits was high in the EHSE environment as compared to the HSE.

High temperatures negatively affect the growth and development of wheat, but the damage depends on the duration of exposure and the developmental stage of the plant exposed to stress (Ruelland and Zachowski, 2010; Balla et al., 2012). A reduction in phenological traits under heat stress is a good indicator for the selection of tolerant plants (Ullah et al., 2022). In the present study, heat-sensitive genotypes showed a significant reduction in phenological traits, namely FLW, DM, PH, NT, SL, SS, GS, TKW, BY, and SY under HSE and EHSE. Days to 50% flowering and maturity are key traits when breeding for late-sown, short-duration, and heat-tolerant wheat cultivars. In our experiments, a significant effect of high temperature was observed for FLW and DM under HSE and EHSE. Under EHSE, a maximum reduction of 28% was observed for FLW. High temperatures induce early flowering (Prasad and Djanaguiraman, 2014), the production of non-viable pollen grains, slow growth of pollen tubes, and failure of fertilization (Saini et al., 1983; Saini et al., 1984; Khan et al., 2021a). The maturity duration of the crop was drastically reduced to 18% under HSE and 36% under EHSE conditions, leading to early senescence and reduced grain weight (Shirdelmoghanloo et al., 2016). The reduction in GS was maximum under EHSE as the crop is exposed to terminal high heat stress, which hampers pollen viability, which in turn increases sterility, ovule viability, successful fertilization, and embryo development (Jäger et al., 2008; Prasad et al., 2008; Kumar et al., 2013; Prasad and Djanaguiraman, 2014). High temperatures during the grain development phase reduce grain filling duration, accelerate maturity, and significantly reduce yield (Chakrabarti et al., 2013). Yield loss was highest in heat-susceptible genotypes (51% under HSE and 81% under EHSE environments). HD 2967 is a mega variety grown over a wide area in the northwestern and northeastern plains of India: it was highly affected by extended heat stress, resulting in an 89% reduction in grain yield. Wheat productivity is adversely affected (3 to 5%) per 1^0^C rise in temperatures through changes in physiology, growth, and yield characteristics (Gibson and Paulsen, 1999).

Physiological traits such as CT, NDVI, CCI, and Chl are the key traits to study genotypes under heat stress environments (Balota et al., 2008; Kumari et al., 2012; Ullah et al., 2019; Ahmed et al., 2020). CT at the heading stage (CT_HS_) was low in heat-tolerant genotypes as compared to heat-sensitive genotypes. Low CT was significantly associated with grain yield in a number of studies (Guo et al., 2016; Kaur et al., 2018; Liang et al., 2018; Sohail et al., 2020). In previous studies, CT was established as a selection criterion as it showed a high correlation with delay in the senescence of leaves (Fang et al., 2017), stomatal conductance (Bonari et al., 2020), leaf and stem wax (Mondal et al., 2015), root system architecture (Pinto and Reynolds, 2015), grain/spike (Sohail et al., 2020), and 1000 grain weight (Gulnaz et al., 2019). Under OE and HSE, CT was negatively associated with phenological and biochemical traits. However, the association of the biochemical and phenological traits with CT was static under EHSE. Thus, CT remained the best selection indicator for heat tolerance under high and extended terminal heat stress. At the grain filling stage, CT was lower in heat-tolerant genotypes, whereas higher CT was observed in heat-sensitive genotypes. The CT_GFS_ was comparatively low in HD 2967, which was associated with high biomass as compared to another sensitive genotype.

High NDVI under high temperatures is positively correlated with grain productivity (Hazratkulova et al., 2012), so NDVI could be used for indirect selection of heat tolerance. However, NDVI readings decreased under EHSE compared to HSE. Despite its declining trend under HSE and EHSE, NDVI had a positive association with seed yield. The experiments showed that the decrease in the NDVI under the EHSE was mainly related to the long-term high-temperature stress during the vegetative and reproductive stages of the crop. In the present study, CCI and Chl were higher in the tolerant genotypes under HSE and EHSE. The heat-tolerant genotypes DBW 150, WH 730, AKW 2862-1, and Halna were found to be superior for maintaining a high chlorophyll content in the flag leaf, while the sensitive genotype lost chlorophyll at high temperatures. High chlorophyll concentrations in plant leaves are a key determinant of high photosynthetic rates under heat stress (Kumari et al., 2012; Roy et al., 2021). Previous studies have also shown that CT, CCI, Chl, and NDVI are associated with heat tolerance and are significantly correlated with SY under high-temperature stress (Pask et al., 2012; Islam et al., 2014). Furthermore, the heat-tolerant cultivars (DBW 150, WH 730, AKW 2862-1, and Halna) showed low CT and high CCI, Chl, and NDVI under heat stress compared to the heat-sensitive genotypes. There were significant differences in the response of different cultivars under HSE to EHSE. The yield decrease in tolerant genotypes was comparatively lower, mainly because they maintained higher GS and TKW than the heat-sensitive genotypes under heat stress. Low CT during grain filling and high CCI, NDVI, and Chl during the reproductive phase in wheat genotypes are indicative of delayed senescence and high photosynthetic rate remobilization of assimilates under terminal heat stress. In conclusion, an extreme heat stress environment decreased the maximum pheno-physiological traits FLW, DM, PH, NT, SL, SS, GS, TKW, BY and CT, NDVI, CCI, and Col, which resulted in low seed yield.

Plants employ both enzymatic and non-enzymatic processes to safeguard themselves from the production of reactive oxygen species (ROS) (Bita and Gerats, 2013). The main enzymes involved in ROS scavenging are superoxide dismutase (SOD), catalase (CAT), peroxidase (POD), ascorbate peroxidase (APX), and glutathione reductase (GR), while the non-enzymatic system includes ascorbic acid (ASC) and glutathione (GSH) (Suzuki et al., 2012). By assessing the expression levels of these enzymes, genotypes can be selected, favoring those with higher activities among heat-tolerant varieties over heat-sensitive ones (Kumar et al., 2013). Biochemical analysis indicated that the activity of SOD, POX, and APX was induced in tolerant genotypes under heat stress as compared to control conditions, whereas a decrease in activities was observed under extended heat stress. The activities of SOD, POX, and APX showed a decreasing trend under heat and extended heat stress in comparison to controls in susceptible genotypes. SOD is a key ROS-scavenging enzyme that catalyzes the dismutation of superoxide to H_2_O_2_ and O_2_. Thus, increased SOD activity has been correlated with increased protection from the damage associated with oxidative stress (Kaur et al., 2021). The presence of a better antioxidant enzyme system, as evidenced by the higher activities of SOD, POX, and APX in heat-tolerant wheat genotypes, could be an indicator that these genotypes are more efficient at scavenging superoxide anions produced in plants due to heat stress and provides a plausible explanation for their tolerant nature under stress conditions. Overexpression of SOD and APX genes, which are utilized in ROS scavenging, has been reported to be one of the mechanisms of enhanced antioxidative defense and ultimately improved tolerance to abiotic stresses (Lee et al., 2007; Hamurcu et al., 2020; Chugh et al., 2022; Sharma et al., 2023). Rajjou and Debeaujon (2008) suggested that a well-developed detoxification mechanism due to the higher activity of antioxidant enzymes in dry seeds helps to preserve their high germination ability. In a recent study, Sehar et al. (2023) reported ethylene-enhanced proline accumulation and an antioxidant defense system, resulting in improved photosynthetic performance and heat stress tolerance in wheat plants. Several other researchers have also reported differential antioxidant responses in contrasting wheat cultivars under terminal heat stress (Yadav et al., 2022; Alsamadany et al., 2023; Kumar et al., 2023b).

Both tolerant and susceptible genotypes accumulated higher proline levels under heat stress conditions as compared to control samples. However, genotypic variation in proline synthesis could be observed under extended heat stress, as tolerant genotypes exhibited a moderate increase in proline levels under extended heat conditions as compared to sensitive genotypes. Since the protective role of proline in the stabilization of proteins and antioxidant enzymes, direct scavenging of ROS, the balance of intracellular redox homeostasis (e.g., NADP^+^/NADPH and GSH/GSSG ratio), and cellular signaling is well documented, the higher content of proline in tolerant wheat genotypes could have an impact on plant survival under heat stress conditions and also serve as a biochemical marker for the early screening of the genotypes (Liang et al., 2013; Dikilitas et al., 2020; Zhang et al., 2022; Sihag et al., 2023). Many plants accumulate a number of compatible osmolytes such as proline and various sugars under drought, heat, salinity, flood, heavy metals, and cold stress conditions, and these osmolytes function as osmo-protectants in plant stress tolerance (Gupta and Kaur, 2005; Chaudhary et al., 2020; Moukhtari et al., 2020; Khan et al., 2021b; Chugh et al., 2022).

Regression analysis in three environments revealed the dynamic relationship between phenological-physio-biochemical traits. In our study, traits BY, DM, CT, NDVI, Chl, and biochemical parameters, particularly SOD, POX, APX, and proline, were observed to play a crucial role in providing tolerance under stress conditions and eventually maintaining seed yield. The relationship between these variables changed in response to different levels of stress environments. This suggests that these traits may respond together to provide tolerance. In conclusion, these traits may help crops better adapt to high-stress conditions and may play a promising role in maintaining yield in such challenging environments. The tolerant genotypes identified in this study could be used for breeding heat-tolerant plants and to develop mapping populations to understand and dissect the underlying genes/QTLs conferring tolerance.

## Data availability statement

The original contributions presented in the study are included in the article/**Supplementary Material**. Further inquiries can be directed to the corresponding authors.

## Author contributions

Conceptualization, HK, VG, SK, and MaK; methodology, HK and MaK; open field experiments and data compilation, HK; biochemical analysis, VC and MaK; software, SP, and CS; validation, HK, SK, and SP; formal analysis, HK and VG; resources, MaK and BS; data curation, SK; writing—original draft preparation, HK and VC; writing—review and editing, RK, MuK, GP, SP, and BS; visualization, RK. All authors contributed to the article and approved the submitted version.
