# Supplementary material for: Investigating the impact of terminal heat stress on contrasting wheat cultivars: a comprehensive analysis of phenological, physiological, and biochemical traits
Source: Front Plant Sci. 2023 Aug 30;14:1189005. doi: 10.3389/fpls.2023.1189005 (PMC10499387; doi:10.3389/fpls.2023.1189005)
Supplement: Supplementary file 1 [file Table_1.docx]

**Appendix A:**

**Tables A1:** The values represent days to 50% Flowering at three environment conditions: OE, HSE and EHSE. The same letter of on different cultivar represent non-significant differences between the cultivars. The reduction percentage represent the values of % reduction from OE to HSE and EHSE.

| **Days to 50% Flowering** | | | | | |
| --- | --- | --- | --- | --- | --- |
| **Cultivar** | **OE** | **HSE** | **EHSE** | **Reduction (%) OE-HSE** | **Reduction (%) OE-EHSE** |
| DBW 150 (HT) | 79^de^ | 66^bc^ | 58^ab^ | 17 | 26 |
| WH 730 (HT) | 84^bc^ | 68^ab^ | 60^a^ | 18 | 29 |
| AKW 2862-1 (HT) | 77^e^ | 69^ab^ | 56^b^ | 10 | 27 |
| Halna (HT) | 63^f^ | 54^d^ | 48^c^ | 14 | 24 |
| K 1006 (HS) | 85^bc^ | 66^bc^ | 59^ab^ | 22 | 30 |
| PBW 550 (HS) | 82^cd^ | 63^c^ | 59^ab^ | 23 | 28 |
| WH 1105(HS) | 86^b^ | 69^ab^ | 61^a^ | 20 | 29 |
| HD 2967(HS) | 90^a^ | 71^a^ | 61^a^ | 21 | 32 |
| **Mean** | **81** | **66** | **58** | **18** | **28** |

**Tables A2:** The values represent days to maturity at three environment conditions: OE, HSE and EHSE. The same letter of on different cultivar represents non-significant differences between the cultivars. The reduction percentage represents the values of % reduction from OE to HSE and EHSE.

| **Days to Maturity** | | | | | |
| --- | --- | --- | --- | --- | --- |
| **Cultivar** | **OE** | **HSE** | **EHSE** | **Reduction (%) OE-HSE** | **Reduction (%) OE-EHSE** |
| DBW 150 (HT) | 136^c^ | 111^a^ | 86^b^ | 18 | 37 |
| WH 730 (HT) | 134^c^ | 111^a^ | 84^bc^ | 17 | 38 |
| AKW 2862-1 (HT) | 134^c^ | 113^a^ | 84^b^ | 16 | 37 |
| Halna (HT) | 120^d^ | 101^b^ | 80^c^ | 15 | 33 |
| K 1006 (HS) | 138^bc^ | 111^a^ | 90^a^ | 19 | 34 |
| PBW 550 (HS) | 137^bc^ | 113^a^ | 90^a^ | 18 | 35 |
| WH 1105(HS) | 140^ab^ | 111^a^ | 85^b^ | 20 | 39 |
| HD 2967(HS) | 143^a^ | 113^a^ | 87^ab^ | 21 | 39 |
| **Mean** | **136** | **111** | **86** | **18** | **36** |

**Tables A3:** The values representplant height (cm)at three environment conditions: OE, HSE and EHSE. The same letter of on different cultivar represents non-significant differences between the cultivars. The reduction percentage represents the values of % reduction from OE to HSE and EHSE.

| **Plant Height (cm)** | | | | | |
| --- | --- | --- | --- | --- | --- |
| **Cultivar** | **OE** | **HSE** | **EHSE** | **Reduction (%) OE-HSE** | **Reduction (%) OE-EHSE** |
| DBW 150 (HT) | 110^c^ | 109^c^ | 73^de^ | 1 | 33 |
| WH 730 (HT) | 124^a^ | 123^a^ | 85^b^ | 1 | 31 |
| AKW 2862-1 (HT) | 120^ab^ | 115^ab^ | 96^a^ | 5 | 20 |
| Halna (HT) | 81^f^ | 75^e^ | 72^bc^ | 8 | 11 |
| K 1006 (HS) | 112^cd^ | 109^bc^ | 80^bcd^ | 3 | 29 |
| PBW 550 (HS) | 96^e^ | 92^d^ | 76^cde^ | 4 | 21 |
| WH 1105(HS) | 100^de^ | 95^d^ | 78^bcd^ | 5 | 22 |
| HD 2967(HS) | 116^bc^ | 110^abc^ | 67^e^ | 5 | 42 |
| **Mean** | **107** | **104** | **78** | **4** | **26** |

**Tables A4:** The values represent number of tillers per plant at three environment conditions: OE, HSE and EHSE. The same letter of on different cultivar represents non-significant differences between the cultivars. The reduction percentage represents the values of % reduction from OE to HSE and EHSE.

| **Number of Tillers per Plant** | | | | | |
| --- | --- | --- | --- | --- | --- |
| **Cultivar** | **OE** | **HSE** | **EHSE** | **Reduction (%) OE-HSE** | **Reduction (%) OE-EHSE** |
| DBW 150 (HT) | 13^bcd^ | 12^a^ | 9^a^ | 12 | 35 |
| WH 730 (HT) | 11^cd^ | 11^ab^ | 10^a^ | 5 | 14 |
| AKW 2862-1 (HT) | 10^d^ | 9^ab^ | 7^a^ | 15 | 30 |
| Halna (HT) | 10^d^ | 8^ab^ | 7^a^ | 20 | 35 |
| K 1006 (HS) | 17^ab^ | 11^ab^ | 8^a^ | 36 | 55 |
| PBW 550 (HS) | 15^abc^ | 10^ab^ | 9^a^ | 34 | 41 |
| WH 1105(HS) | 13^cd^ | 8^b^ | 8^a^ | 36 | 40 |
| HD 2967(HS) | 17^a^ | 11^ab^ | 7^a^ | 38 | 62 |
| **Mean** | **13** | **10** | **8** | **25** | **39** |

**Tables A5:** The values represent spike length (cm)at three environment conditions: OE, HSE and EHSE. The same letter of on different cultivar represents non-significant differences between the cultivars. The reduction percentage represents the values of % reduction from OE to HSE and EHSE.

| **Spike Length (cm)** | | | | | |
| --- | --- | --- | --- | --- | --- |
| **Cultivar** | **OE** | **HSE** | **EHSE** | **Reduction (%) OE-HSE** | **Reduction (%) OE-EHSE** |
| DBW 150 (HT) | 11^a^ | 10^ab^ | 8^cd^ | 6 | 29 |
| WH 730 (HT) | 12^a^ | 12^a^ | 11^a^ | 1 | 9 |
| AKW 2862-1 (HT) | 11^ab^ | 10^bc^ | 10^ab^ | 3 | 3 |
| Halna (HT) | 9^c^ | 8^c^ | 8^bcd^ | 2 | 2 |
| K 1006 (HS) | 11^ab^ | 10^ab^ | 9^bc^ | 4 | 21 |
| PBW 550 (HS) | 9^bc^ | 8^c^ | 7^d^ | 9 | 28 |
| WH 1105(HS) | 11^a^ | 10^bc^ | 10^abc^ | 11 | 14 |
| HD 2967(HS) | 12^a^ | 11^ab^ | 9^bc^ | 8 | 20 |
| **Mean** | **11** | **10** | **9** | **6** | **16** |

**Tables A6:** The values represent number of spikelet/spike at three environment conditions: OE, HSE and EHSE. The same letter of on different cultivar represents non-significant differences between the cultivars. The reduction percentage represents the values of % reduction from OE to HSE and EHSE.

| **Number of Spikelet /Spike** | | | | | |
| --- | --- | --- | --- | --- | --- |
| **Cultivar** | **OE** | **HSE** | **EHSE** | **Reduction (%) OE-HSE** | **Reduction (%) OE-EHSE** |
| DBW 150 (HT) | 23^bc^ | 21^abc^ | 19^c^ | 9 | 20 |
| WH 730 (HT) | 25^a^ | 20^bc^ | 21^a^ | 20 | 18 |
| AKW 2862-1 (HT) | 22^c^ | ^20ab^ | 19^a^ | 9 | 14 |
| Halna (HT) | 21^d^ | 19^bc^ | 17^c^ | 7 | 20 |
| K 1006 (HS) | 22^c^ | 20^bc^ | 17^c^ | 9 | 25 |
| PBW 550 (HS) | 17^e^ | 17^d^ | 14^bc^ | 0 | 15 |
| WH 1105(HS) | 22^c^ | 20^c^ | 19^b^ | 9 | 14 |
| HD 2967(HS) | 24^ab^ | 23^a^ | 17^c^ | 6 | 31 |
| **Mean** | **22** | **20** | **17** | **9** | **20** |

**Tables A7:** The values represent grain number\spike at three environment conditions: OE, HSE and EHSE. The same letter of on different cultivar represents non-significant differences between the cultivars. The reduction percentage represents the values of % reduction from OE to HSE and EHSE.

| **Grain Number\Spike** | | | | | |
| --- | --- | --- | --- | --- | --- |
| **Cultivar** | **OE** | **HSE** | **EHSE** | **Reduction (%) OE-HSE** | **Reduction (%) OE-EHSE** |
| DBW 150 (HT) | 70^a^ | 55^ab^ | 48^b^ | 21 | 31 |
| WH 730 (HT) | 61^b^ | 52^ab^ | 51^a^ | 15 | 16 |
| AKW 2862-1 (HT) | 61^b^ | 58^a^ | 38^bcd^ | 5 | 38 |
| Halna (HT) | 56^b^ | 54^a^ | 43^bc^ | 4 | 23 |
| K 1006 (HS) | 61^b^ | 42^c^ | 35^cd^ | 31 | 43 |
| PBW 550 (HS) | 54^b^ | 47^bc^ | 37^bcd^ | 13 | 31 |
| WH 1105(HS) | 55^b^ | 48^bc^ | 29^d^ | 13 | 47 |
| HD 2967(HS) | 54^b^ | 50^a^ | 40^bc^ | 7 | 26 |
| **Mean** | **59** | **51** | **40** | **14** | **32** |

**Tables A8:** The values represent test kernel weight (gm) at three environment conditions: OE, HSE and EHSE. The same letter of on different cultivar represents non-significant differences between the cultivars. The reduction percentage represents the values of % reduction from OE to HSE and EHSE.

| **Test Kernel Weight (gm)** | | | | | |
| --- | --- | --- | --- | --- | --- |
| **Cultivar** | **OE** | **HSE** | **EHSE** | **Reduction (%) OE-HSE** | **Reduction (%) OE-EHSE** |
| DBW 150 (HT) | 3.89^c^ | 3.67^bc^ | 3.64^a^ | 5.45 | 6.42 |
| WH 730 (HT) | 4.12^bc^ | 3.61^bc^ | 3.05^b^ | 12.39 | 26.03 |
| AKW 2862-1 (HT) | 4.97^a^ | 4.16^a^ | 3.52^a^ | 16.20 | 29.14 |
| Halna (HT) | 4.31^b^ | 3.75^bc^ | 3.43^c^ | 13.08 | 20.41 |
| K 1006 (HS) | 3.96^bc^ | 3.21^cd^ | 2.81^a^ | 18.89 | 28.94 |
| PBW 550 (HS) | 4.33^b^ | 3.02^d^ | 2.85^a^ | 30.25 | 34.18 |
| WH 1105(HS) | 4.10^bc^ | 3.07^ab^ | 2.54^c^ | 25.06 | 38.10 |
| HD 2967(HS) | 3.89^c^ | 3.15^c^ | 2.25^b^ | 18.96 | 42.31 |
| **Mean** | **4.19** | **3.46** | **3.05** | **17.54** | **27.33** |

**Tables A9:** The values represent biological yield(kg)/plot at three environment conditions: OE, HSE and EHSE. The same letter of on different cultivar represents non-significant differences between the cultivars. The reduction percentage represents the values of % reduction from OE to HSE and EHSE.

| **Biological Yield(kg)/Plot** | | | | | |
| --- | --- | --- | --- | --- | --- |
| **Cultivar** | **OE** | **HSE** | **EHSE** | **Reduction (%) OE-HSE** | **Reduction (%) OE-EHSE** |
| DBW 150 (HT) | 2.15^a^ | 1.78^a^ | 1.15^a^ | 17.07 | 46.48 |
| WH 730 (HT) | 2.24^a^ | 1.85^ab^ | 1.07^a^ | 17.39 | 52.31 |
| AKW 2862-1 (HT) | 1.98^a^ | 1.86^ab^ | 1.25^a^ | 6.22 | 37.24 |
| Halna (HT) | 1.49^c^ | 1.11^b^ | 0.99^a^ | 25.71 | 33.68 |
| K 1006 (HS) | 1.54^bc^ | 1.21^ab^ | 0.74^a^ | 21.29 | 52.05 |
| PBW 550 (HS) | 1.28^c^ | 1.10^ab^ | 0.77^a^ | 14.18 | 39.52 |
| WH 1105(HS) | 1.59^bc^ | 1.29^ab^ | 0.70^a^ | 18.61 | 56.06 |
| HD 2967(HS) | 2.13^ab^ | 1.25^ab^ | 0.61^a^ | 41.17 | 71.48 |
| **Mean** | **1.80** | **1.43** | **0.91** | **20.21** | **48.60** |

**Tables A10:** The values represent seed yield (kg)/plot at three environment conditions: OE, HSE and EHSE. The same letter of on different cultivar represents non-significant differences between the cultivars. The reduction percentage represents the values of % reduction from OE to HSE and EHSE.

| **Seed Yield (kg)/plot** | | | | | |
| --- | --- | --- | --- | --- | --- |
| **Cultivar** | **OE** | **HSE** | **EHSE** | **Reduction (%) OE-HSE** | **Reduction (%) OE-EHSE** |
| DBW 150 (HT) | 1.10^ab^ | 0.74^ab^ | 0.45^ab^ | 32.58 | 59.67 |
| WH 730 (HT) | 1.19^a^ | 0.82^a^ | 0.54^ab^ | 31.61 | 54.93 |
| AKW 2862-1 (HT) | 0.91^c^ | 0.70^a^ | 0.54^ab^ | 22.61 | 40.32 |
| Halna (HT) | 0.77^d^ | 0.56^ab^ | 0.42^a^ | 27.49 | 45.41 |
| K 1006 (HS) | 0.82^d^ | 0.46^ab^ | 0.19^ab^ | 44.12 | 77.21 |
| PBW 550 (HS) | 0.92^d^ | 0.42^b^ | 0.19^ab^ | 54.52 | 79.16 |
| WH 1105(HS) | 0.97^bc^ | 0.43^b^ | 0.22^ab^ | 55.64 | 77.07 |
| HD 2967(HS) | 1.15^abc^ | 0.57^ab^ | 0.12^b^ | 50.63 | 89.54 |
| **Mean** | **0.98** | **0.59** | **0.33** | **39.90** | **65.41** |

**Tables A11:** The values represent canopy temperature at heading stage at three environment conditions: OE, HSE and EHSE. The same letter of on different cultivar represents non-significant differences between the cultivars. The reduction percentage represents the values of % reduction from OE to HSE and EHSE.

| **Canopy Temperature at Heading Stage** | | | | | |
| --- | --- | --- | --- | --- | --- |
| **Cultivar** | **OE** | **HSE** | **EHSE** | **Reduction (%) OE-HSE** | **Reduction (%) OE-EHSE** |
| DBW 150 (HT) | 19.92^c^ | 19.25^b^ | 30.17^b^ | 3.36 | -51.43 |
| WH 730 (HT) | 19.83^c^ | 19.47^b^ | 31.34^b^ | 1.84 | -58.02 |
| AKW 2862-1 (HT) | 20.97^c^ | 17.79^c^ | 30.12^d^ | 15.19 | -43.61 |
| Halna (HT) | 23.60^ab^ | 18.95^bc^ | 30.20^d^ | 19.70 | -27.97 |
| K 1006 (HS) | 23.39a^b^ | 21.70^a^ | 34.27^b^ | 7.21 | -46.53 |
| PBW 550 (HS) | 24.25^a^ | 21.77^a^ | 33.75^c^ | 10.25 | -39.18 |
| WH 1105(HS) | 22.65^b^ | 22.69^a^ | 36.57^a^ | -0.15 | -61.43 |
| HD 2967(HS) | 19.93^c^ | 19.55^b^ | 33.54^d^ | 1.93 | -68.26 |
| **Mean** | **21.82** | **20.14** | **32.49** | **7.42** | **-49.55** |

**Tables A12:** The values represent canopy temperature at mid gain-filling stage at three environment conditions: OE, HSE and EHSE. The same letter of on different cultivar represents non-significant differences between the cultivars. The reduction percentage represents the values of % reduction from OE to HSE and EHSE.

| **Canopy Temperature at Mid Gain-filling Stage** | | | | | |
| --- | --- | --- | --- | --- | --- |
| **Cultivar** | **OE** | **HSE** | **EHSE** | **Reduction (%) OE-HSE** | **Reduction (%) OE-EHSE** |
| DBW 150 (HT) | 21.05^bc^ | 31.60^bc^ | 45.42^a^ | -50.12 | -115.75 |
| WH 730 (HT) | 19.50^c^ | 32.27^b^ | 44.25^a^ | -65.46 | -126.92 |
| AKW 2862-1 (HT) | 22.55^b^ | 28.79^d^ | 41.59^b^ | -27.65 | -84.41 |
| Halna (HT) | 24.82^a^ | 29.87^cd^ | 44.82^a^ | -20.35 | -80.60 |
| K 1006 (HS) | 24.85^a^ | 33.25^ab^ | 34.24^d^ | -33.80 | -37.77 |
| PBW 550 (HS) | 25.04^a^ | 34.84^a^ | 33.92^d^ | -39.15 | -35.47 |
| WH 1105(HS) | 24.47^a^ | 34.15^a^ | 38.00^c^ | -39.56 | -55.29 |
| HD 2967(HS) | 24.39^a^ | 30.2^cd^ | 44.57^a^ | -23.85 | -82.76 |
| **Mean** | **23.33** | **31.87** | **40.85** | **-37.49** | **-77.37** |

**Tables A13:** The values represent normalized difference vegetation index at heading stage at three environment conditions: OE, HSE and EHSE. The same letter of on different cultivar represents non-significant differences between the cultivars. The reduction percentage represents the values of % reduction from OE to HSE and EHSE.

| **Normalized Difference Vegetation Index at Heading Stage** | | | | | |
| --- | --- | --- | --- | --- | --- |
| **Cultivar** | **OE** | **HSE** | **EHSE** | **Reduction (%) OE-HSE** | **Reduction (%) OE-EHSE** |
| DBW 150 (HT) | 642^a^ | 625^ab^ | 426^d^ | 3 | 34 |
| WH 730 (HT) | 656^a^ | 605^ab^ | 505^ab^ | 8 | 23 |
| AKW 2862-1 (HT) | 657^a^ | 615^a^ | 502^ab^ | 6 | 24 |
| Halna (HT) | 560^b^ | 525^b^ | 486^abc^ | 6 | 13 |
| K 1006 (HS) | 640^a^ | 560^ab^ | 436^abc^ | 12 | 32 |
| PBW 550 (HS) | 554^b^ | 525^ab^ | 429^bcd^ | 5 | 23 |
| WH 1105(HS) | 631^a^ | 572^a^ | 413^cd^ | 9 | 35 |
| HD 2967(HS) | 654^a^ | 554^ab^ | 490^a^ | 15 | 25 |
| **Mean** | **624** | **573** | **461** | **8** | **26** |

**Tables A14:** The values represent normalized difference vegetation index at mid gain-filling stage at three environment conditions: OE, HSE and EHSE. The same letter of on different cultivar represents non-significant differences between the cultivars. The reduction percentage represents the values of % reduction from OE to HSE and EHSE.

| **Normalized Difference Vegetation Index at Mid Gain-filling Stage** | | | | | |
| --- | --- | --- | --- | --- | --- |
| **Cultivar** | **OE** | **HSE** | **EHSE** | **Reduction (%) OE-HSE** | **Reduction (%) OE-EHSE** |
| DBW 150 (HT) | 575^ab^ | 507^a^ | 334^d^ | 12 | 42 |
| WH 730 (HT) | 586^a^ | 526^a^ | 464^bc^ | 10 | 21 |
| AKW 2862-1 (HT) | 571^ab^ | 536^a^ | 478^bc^ | 6 | 16 |
| Halna (HT) | 569^b^ | 456^a^ | 308^d^ | 20 | 46 |
| K 1006 (HS) | 542^ab^ | 515^a^ | 385^a^ | 5 | 29 |
| PBW 550 (HS) | 501^ab^ | 434^a^ | 346^ab^ | 13 | 31 |
| WH 1105(HS) | 553^ab^ | 423^a^ | 345^c^ | 24 | 38 |
| HD 2967(HS) | 583^a^ | 561^a^ | 315^abc^ | 4 | 46 |
| **Mean** | **560** | **494** | **372** | **12** | **34** |

**Tables A15:** The values represent chlorophyll concentration index (cci) at mid gain-filling stage at three environment conditions: OE, HSE and EHSE. The same letter of on different cultivar represents non-significant differences between the cultivars. The reduction percentage represents the values of % reduction from OE to HSE and EHSE.

| **Chlorophyll Concentration Index (CCI) at Mid Gain-filling Stage** | | | | | |
| --- | --- | --- | --- | --- | --- |
| **Cultivar** | **OE** | **HSE** | **EHSE** | **Reduction (%) OE-HSE** | **Reduction (%) OE-EHSE** |
| DBW 150 (HT) | 35.85^b^ | 30.95^cd^ | 27.75^c^ | 13.67 | 22.59 |
| WH 730 (HT) | 36.00^cd^ | 32.00^bc^ | 28.45^b^ | 61.11 | 20.97 |
| AKW 2862-1 (HT) | 35.30^b^ | 30.00^a^ | 28.65^d^ | 15.01 | 18.84 |
| Halna (HT) | 38.00^a^ | 35.00^ab^ | 32.00^a^ | 7.89 | 15.79 |
| K 1006 (HS) | 33.00^cd^ | 27.00^d^ | 24.00^c^ | 18.18 | 27.27 |
| PBW 550 (HS) | 30.65^cd^ | 24.00^d^ | 22.00^cd^ | 21.70 | 28.22 |
| WH 1105(HS) | 31.00^bc^ | 28.00^a^ | 24.00^c^ | 9.68 | 22.58 |
| HD 2967(HS) | 32.00^d^ | 30.10^d^ | 21.55^c^ | 5.94 | 32.66 |
| **Mean** | **33.98** | **29.63** | **25.55** | **19.15** | **23.62** |

**Tables A15:** The values represent chlorophyll concentration (µmol m2) at mid gain-filling stage at three environment conditions: OE, HSE and EHSE. The same letter of on different cultivar represents non-significant differences between the cultivars. The reduction percentage represents the values of % reduction from OE to HSE and EHSE.

| **Chlorophyll Concentration (µmol m2) at Mid Gain-filling Stage** | | | | | |
| --- | --- | --- | --- | --- | --- |
| **Cultivar** | **OE** | **HSE** | **EHSE** | **Reduction (%) OE-HSE** | **Reduction (%) OE-EHSE** |
| DBW 150 (HT) | 580.50^ab^ | 469.20^bc^ | 429.25^bc^ | 19.17 | 26.06 |
| WH 730 (HT) | 607.30^a^ | 524.55^abc^ | 478.45^c^ | 13.63 | 21.22 |
| AKW 2862-1 (HT) | 600.60^ab^ | 548.80^ab^ | 417.85^bc^ | 8.62 | 30.43 |
| Halna (HT) | 568.30^ab^ | 558.85^a^ | 463.45^a^ | 1.66 | 18.45 |
| K 1006 (HS) | 511.00^c^ | 411.40^abc^ | 376.50^b^ | 19.49 | 26.32 |
| PBW 550 (HS) | 516.95^b^ | 441.85^c^ | 333.55^bc^ | 14.53 | 35.48 |
| WH 1105(HS) | 538.25^ab^ | 452.45^c^ | 388.00^d^ | 15.94 | 27.91 |
| HD 2967(HS) | 556.60^ab^ | 438.65^ab^ | 304.20^bc^ | 21.19 | 45.35 |
| **Mean** | **559.94** | **480.72** | **398.91** | **14.28** | **28.90** |

**Tables A16:** The values represent SOD value at three environment conditions: OE, HSE and EHSE. The same letter of on different cultivar represents non-significant differences between the cultivars. The reduction percentage represents the values of % reduction from OE to HSE and EHSE.

| **SOD** | | | | | |
| --- | --- | --- | --- | --- | --- |
| **Cultivar** | **OE** | **HSE** | **EHSE** | **Reduction (%) OE-HSE** | **Reduction (%) OE-EHSE** |
| DBW 150 (HT) | 84.30^d^ | 104.41^c^ | 74.75^a^ | -23.85 | 11.33 |
| WH 730 (HT) | 93.65^b^ | 114.72^b^ | 66.35^b^ | -22.50 | 29.15 |
| AKW 2862-1 (HT) | 109.70^a^ | 146.24^a^ | 73.30^a^ | -33.31 | 33.18 |
| Halna (HT) | 90.75^bc^ | 100.56^c^ | 79.10^a^ | -10.82 | 12.84 |
| K 1006 (HS) | 82.85^d^ | 66.31e | 53.18^c^ | 19.96 | 35.81 |
| PBW 550 (HS) | 81.52^de^ | 60.17^f^ | 45.36^d^ | 26.19 | 44.36 |
| WH 1105(HS) | 76.70^e^ | 68.10^e^ | 41.53^d^ | 11.21 | 45.85 |
| HD 2967(HS) | 86.69^cd^ | 81.15^d^ | 62.50^b^ | 6.39 | 27.91 |
| **Mean** | **88.27** | **92.71** | **62.01** | **-3.34** | **30.05** |

**Tables A17:** The values represent POX value at three environment conditions: OE, HSE and EHSE. The same letter of on different cultivar represents non-significant differences between the cultivars. The reduction percentage represents the values of % reduction from OE to HSE and EHSE.

| **POX** | | | | | |
| --- | --- | --- | --- | --- | --- |
| **Cultivar** | **OE** | **HSE** | **EHSE** | **Reduction (%) OE-HSE** | **Reduction (%) OE-EHSE** |
| DBW 150 (HT) | 21.37^cd^ | 30.21^c^ | 19.10^a^ | -41.36 | 10.64 |
| WH 730 (HT) | 23.75^b^ | 32.09^b^ | 16.72^b^ | -35.14 | 29.58 |
| AKW 2862-1 (HT) | 27.57^a^ | 36.85^a^ | 18.58^a^ | -33.64 | 32.62 |
| Halna (HT) | 23.01^bc^ | 25.40^d^ | 20.08^a^ | -10.37 | 12.72 |
| K 1006 (HS) | 21.26^cde^ | 16.86^f^ | 13.34^c^ | 20.68 | 37.24 |
| PBW 550 (HS) | 20.46^de^ | 15.42^f^ | 11.25^de^ | 24.66 | 44.99 |
| WH 1105(HS) | 19.57^e^ | 17.12^f^ | 10.50^e^ | 12.52 | 46.33 |
| HD 2967(HS) | 21.49^cd^ | 20.03^e^ | 12.47^cd^ | 6.78 | 41.95 |
| **Mean** | **22.31** | **24.25** | **15.26** | **-6.98** | **32.01** |

**Tables A18:** The values represent APX value at three environment conditions: OE, HSE and EHSE. The same letter of on different cultivar represents non-significant differences between the cultivars. The reduction percentage represents the values of % reduction from OE to HSE and EHSE.

| **APX** | | | | | |
| --- | --- | --- | --- | --- | --- |
| **Cultivar** | **OE** | **HSE** | **EHSE** | **Reduction (%) OE-HSE** | **Reduction (%) OE-EHSE** |
| DBW 150 (HT) | 784.78^cd^ | 2299.77^a^ | 1072.85^a^ | -193.05 | -36.71 |
| WH 730 (HT) | 762.98^d^ | 1615.32^b^ | 547.25^d^ | -111.71 | 28.27 |
| AKW 2862-1 (HT) | 837.08^b^ | 1310.88^d^ | 615.79^c^ | -56.60 | 26.44 |
| Halna (HT) | 764.29^d^ | 1431.43^c^ | 683.13^b^ | -87.29 | 10.62 |
| K 1006 (HS) | 798.30^c^ | 832.86^g^ | 340.99^g^ | -4.33 | 57.29 |
| PBW 550 (HS) | 759.70^d^ | 617.56^h^ | 375.40^f^ | 18.71 | 50.59 |
| WH 1105(HS) | 888.26^a^ | 1060.52^e^ | 426.04^e^ | -19.39 | 52.04 |
| HD 2967(HS) | 850.85^b^ | 1015.98^f^ | 596.76^c^ | -19.41 | 29.86 |
| **Mean** | **805.78** | **1273.04** | **582.27** | **-59.13** | **27.3** |

**Tables A19:** The values represent CAT value at three environment conditions: OE, HSE and EHSE. The same letter of on different cultivar represents non-significant differences between the cultivars. The reduction percentage represents the values of % reduction from OE to HSE and EHSE.

| **CAT** | | | | | |
| --- | --- | --- | --- | --- | --- |
| **Cultivar** | **OE** | **HSE** | **EHSE** | **Reduction (%) OE-HSE** | **Reduction (%) OE-EHSE** |
| DBW 150 (HT) | 323.48^c^ | 338.14^a^ | 116.27^g^ | -4.53 | 64.06 |
| WH 730 (HT) | 313.51^d^ | 237.49^e^ | 161.28^d^ | 24.25 | 48.56 |
| AKW 2862-1 (HT) | 342.64^b^ | 192.74^f^ | 167.45^c^ | 43.75 | 51.13 |
| Halna (HT) | 312.76^d^ | 310.50^c^ | 174.26^b^ | 0.72 | 44.28 |
| K 1006 (HS) | 326.09^c^ | 323.37^b^ | 138.50^f^ | 0.83 | 57.53 |
| PBW 550 (HS) | 310.85^d^ | 251.67^d^ | 241.41^a^ | 19.04 | 22.34 |
| WH 1105(HS) | 364.57^a^ | 155.90^g^ | 146.29^e^ | 57.24 | 59.87 |
| HD 2967(HS) | 347.09^b^ | 149.35^h^ | 164.73^cd^ | 56.97 | 52.54 |
| **Mean** | **330.13** | **244.89** | **163.77** | **24.78** | **50.04** |

**Tables A20:** The values represent Proline value at three environment conditions: OE, HSE and EHSE. The same letter of on different cultivar represents non-significant differences between the cultivars. The reduction percentage represents the values of % reduction from OE to HSE and EHSE.

| **Proline** | | | | | |
| --- | --- | --- | --- | --- | --- |
| **Cultivar** | **OE** | **HSE** | **EHSE** | **Reduction (%) OE-HSE** | **Reduction (%) OE-EHSE** |
| DBW 150 (HT) | 0.09^cd^ | 0.15^a^ | 0.10^d^ | -71.29 | -14.00 |
| WH 730 (HT) | 0.09^b^ | 0.14^b^ | 0.11^b^ | -43.22 | -15.36 |
| AKW 2862-1 (HT) | 0.11^a^ | 0.15^a^ | 0.13^a^ | -31.86 | -15.07 |
| Halna (HT) | 0.09^b^ | 0.11^c^ | 0.10^c^ | -21.2 | -11.04 |
| K 1006 (HS) | 0.08^d^ | 0.10^d^ | 0.08^e^ | -14.35 | 10.29 |
| PBW 550 (HS) | 0.08^d^ | 0.09^e^ | 0.07^f^ | -8.51 | 17.98 |
| WH 1105(HS) | 0.08^e^ | 0.09^e^ | 0.07^e^ | -13.05 | 5.04 |
| HD 2967(HS) | 0.09^c^ | 0.10^d^ | 0.07^f^ | -12.44 | 25.34 |
| **Mean** | **0.09** | **0.114** | **0.09** | **-26.99** | **0.4** |
